# Impact and cost-effectiveness of a universal strategy to promote physical activity in primary care: population-based Cohort study and Markov model

**DOI:** 10.1007/s10198-013-0477-0

**Published:** 2013-04-10

**Authors:** Martin C. Gulliford, Judith Charlton, Nawaraj Bhattarai, Christopher Charlton, Caroline Rudisill

**Affiliations:** 1 Department of Primary Care and Public Health Sciences, King’s College London, Capital House, 42 Weston St, London, SE1 3QD UK; 2 Centre for Multilevel Modelling, University of Bristol, Bristol, UK; 3 Department of Social Policy, London School of Economics and Political Science, London, UK

**Keywords:** Physical activity, Primary care, Markov model, Outcomes, Cost-effectiveness, Depression, Diabetes, Coronary heart disease, Stroke, Colorectal cancer, I10 Health, General, D61, Allocative efficiency

## Abstract

**Background:**

This study aimed to estimate the cost-effectiveness of a universal strategy to promote physical activity in primary care.

**Methods:**

Data were analysed for a cohort of participants from the general practice research database. Empirical estimates informed a Markov model that included five long-term conditions (diabetes, coronary heart disease, stroke, colorectal cancer and depression). Simulations compared an intervention promoting physical activity in healthy adults with standard care. The intervention effect on physical activity was from a meta-analysis of randomised trials. The annual cost of intervention, in the base case, was one family practice consultation per participant year. The primary outcome was net health benefit in quality adjusted life years (QALYs).

**Results:**

A cohort of 262,704 healthy participants entered the model. Intervention was associated with an increase in life years lived free from physical disease. With 5 years intervention the increase was 52 (95 % interval −11 to 115) per 1,000 participants entering the model (probability increased 91.9 %); with 10 years intervention the increase was 102 (42–164) per 1,000 (probability 99.7 %). Net health benefits at a threshold of £30,000 per QALY were 3.2 (−11.1 to 16.9) QALYs per 1,000 participants with 5 years intervention (probability cost-effective 64.7 %) and 5.0 (−9.5 to 19.3) with 10 years intervention (probability cost-effective 72.4 %).

**Conclusions:**

A universal strategy to promote physical activity in primary care has the potential to increase life years lived free from physical disease. There is only weak evidence that a universal intervention strategy might prove cost-effective.

## Introduction

Physical inactivity is one of the most important risk factors for chronic disease [1]. Epidemiological studies show that higher levels of physical activity are associated with lower frequency of diabetes, coronary heart disease, stroke, colorectal cancer and depression [1]. The promotion of physical activity has become a key global public health objective [2]. Current recommendations recognise that there is a ‘clear link between physical activity and chronic disease’ [3] and advise that all adults should take at least 150 min of moderate physical activity per week with daily activity. Achieving this objective requires action through multiple sectors and at different levels [4]. This research considers the role of primary care services in promoting physical activity.

Health services increasingly emphasise healthy ageing, aiming to prevent disease and reduce the impact of long-term conditions in later life [5]. Primary care services have a potentially important role to play in promoting physical activity. A recent meta-analysis of randomised trials found that brief interventions in primary care could result in one additional person achieving recommended physical activity levels for each 12 persons exposed to the intervention, with the effect being maintained over 12 months [6]. Williams [7] observed that ‘brief exercise advice has a small effect on increasing physical activity… Such a small effect could be important if carried out on a large population of patients’ [7].

The potential long-term effects of interventions to promote physical activity in primary care are not known. Existing reviews have included studies up to 2 years duration with behaviour change and physical fitness as outcomes [6]. Evidence that increased physical activity may reduce the incidence of diabetes is drawn from studies that included high risk individuals with impaired glucose tolerance or pre-diabetes [8]. The present research therefore aimed to evaluate the potential long term health outcomes and cost effectiveness of a universal strategy to promote physical activity in primary care. The research specifically aimed to determine whether a low-cost intervention with a limited intervention effect size, such as that evidenced by the recent meta-analysis on the effectiveness of such interventions [6], would prove cost-effective for implementation across the general population through primary health care services.

## Methods

### Markov model structure

**Fig. 1:**
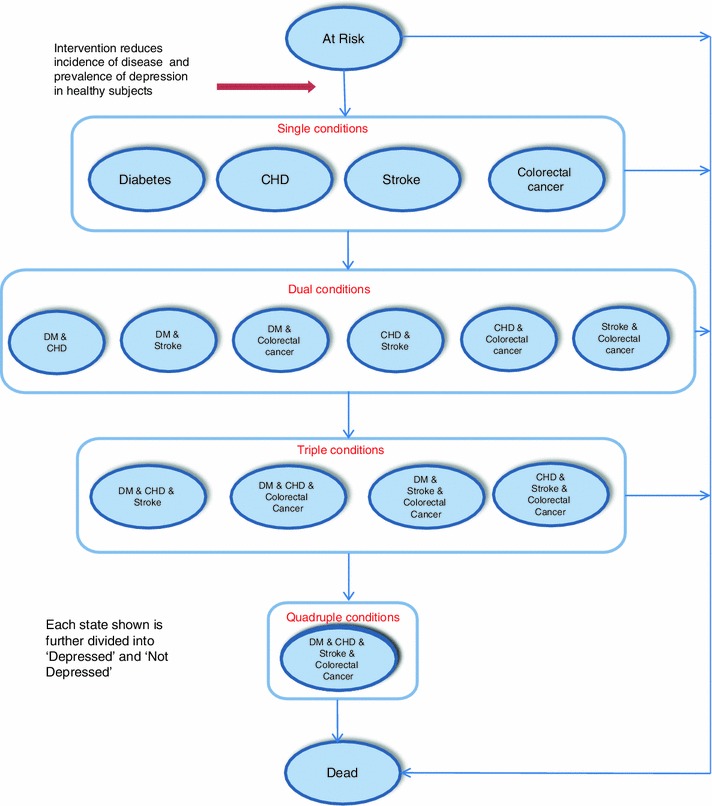
Schematic diagram of Markov model. In order to simplify the diagram, the 32 incidence transitions and 16 mortality transitions are not represented in full. Each state is further divided into ‘depressed’ and ‘not depressed’.* CHD* Coronary heart disease,* DM* type 2 diabetes mellitus

A Markov model was employed to implement a cost-utility analysis of a universal strategy to promote physical activity in the general population registered in primary care, comparing a brief intervention to promote physical activity with ‘standard care’ in which there is no systematic approach to promote physical activity. A simplified diagram of the Markov model is shown in Fig. 1. The model structure was informed by previously reported research [9]. Healthy subjects, referred to as ‘At Risk’, may develop one of the disease states of interest, including Diabetes, Coronary Heart Disease, Stroke or Colorectal Cancer. Participants in one of these disease states may develop a second, third or fourth disease, giving 16 single- or multi-disease states, consistent with the frequent occurrence of multimorbidity as a driver of health care utilisation in primary care populations [10]. Participants in each state were allowed to progress to Depression, with each state divided into states representing ‘Not Depressed’ and ‘Depressed’. Depression was associated with its own decrement in utility as well as its own rate of health care utilisation. Depression was included because it occurs frequently in chronic illness and is associated with higher health care costs for a given chronic illness [11]. There were therefore 32 states in the model that represented all potential combinations of the included diseases and depression. All states might lead to death. The perspective of the model is that of health care services and only health care costs were included. A lifetime time horizon was used.

### GPRD cohort

Data to populate the model were derived from a large cohort of participants drawn from the general population registered with the general practice research database (GPRD) [12]. The GPRD includes electronic health records of participants registered with approximately 600 UK family practices. GPRD data were used to estimate the incidence of each state included in the model; the mortality in each state; and the health care utilisation and costs associated with each state. The use of fully anonymised GPRD data was approved by the MHRA Independent Scientific Advisory Committee (Ref. 09-085).

The GPRD cohort was drawn from all family practices that were continuously contributing data to the GPRD between 1 January 2004 and 30 October 2010. Participants comprised a random sample of 299,912 registered patients, aged 30–100 years. Incidence and mortality rates were estimated, by 10-year age group and sex. As colorectal cancer cases were less numerous, we made the assumptions that the incidence of colorectal cancer was the same in participants with and without cardiovascular comorbidity, and the incidence of cardiovascular comorbidity was the same in participants with and without colorectal cancer. Incidence and mortality rates were similar to those reported from GPRD previously [13–15]. The prevalence of depression was estimated for each state in the Model based on GPRD data [11]. In the Model, we assumed that depression was not associated with mortality at any given level of physical disease.

Health care utilisation was estimated for each state from GPRD records, including utilisation of primary care (family practice consultations, telephone consultations, home visits and emergency and out-of-hours consultations), secondary care (including hospital admissions, outpatient visits, day case visits and emergency visits) and prescriptions. The annual costs associated with each state were estimated by multiplying the health care utilisation associated with the state by the costs of each unit of health care, which were obtained from standard reference sources for 2010 [16] (Table 1). Prescription costs were obtained by linking the Multilex drug code for each prescription record in GPRD with the prescription cost [17]. The empirical mean (standard deviation) of participant level costs were estimated by age group, gender, depression status and condition. As the number of colorectal cancer cases was too small to subdivide into multi-disease states, health care utilisation was estimated for all colorectal cancer cases together.

**Table 1:** Sources of data for model

Data	Number/value	Source	Stratification	Comments
Base population	299,912	GPRD	Gender, 1-year age group	Participants with prevalent disease were excluded. Age range 30–100 years
Model states	33 states		Stratified by gender, 1-year age group.	Includes At Risk, Diabetes, CHD, Stroke, Colorectal cancer, Depression and all combinations, dead
Incidence of states	32 incidence rates	GPRD	Gender, 10-year age group	Beta-binomial distribution used to estimate transition probabilities; incidence assumed independent of depression; note that the same state may be reached by more than one transition (e.g. CHD in diabetes, diabetes in CHD)
Mortality of states	16 mortality rates	GPRD	Gender, 10-year age group	Beta-binomial distribution used to estimate transition probabilities; mortality assumed independent of depression
Depression prevalence	16 depression prevalence rates	GPRD	Gender, 10-year age group	Beta-binomial distribution used to estimate transition probabilities
Health care utilization	Utilisation rates estimated for 32 states	GPRD	Gender, 10-year age group, depression status	Utilisation included primary care consultations (including at general practice, home, telephone and out of hours or emergency), secondary care (including inpatient, outpatient, day case and emergency) and prescription number and content
Unit costs of health care utilization				
Primary care				
Consultation	£35	PSSRU [16]		Gamma distribution used to sample costs
Emergency/out of hours consultation	£35	PSSRU [16]		
Home visit	£117	PSSRU [16]		
Telephone consultation	£21	PSSRU [16]		
Prescription unit costs	Variable	FDBE [16]		Unit price linked by Multilex code to GPRD prescription. Single pack price assumed
Secondary care				
Inpatient episodes	£493	PSSRU [16]		
Outpatient visits	£189	PSSRU [16]		
Day case visits	£143	PSSRU [16]		
Emergency visits	£110	PSSRU [16]		
Utility values	Utility decrement			
Age 43 years	0.828	Sullivan et al. [19]	Stratified by single year of age	Beta function employed to sample utility values
Per year increase in age	−0.00029			
Diabetes	−0.0621			
CHD	−0.0557			
Stroke	−0.1009			
Colorectal cancer	−0.0378			
Depression	−0.1302			
No. of chronic conditions 2	−0.0615			
No. of chronic conditions 3	−0.0667			
No. of chronic conditions 4	−0.0433			
No. of chronic conditions 5	−0.0287			

* GPRD* General practice research database,* PSSRU* Personal Social Services Research Unit,* FDBE* First DataBank Europe

### Model estimation

The probabilistic Markov model was estimated by cohort simulation, implemented through a program written in R software [18]. After removing participants with prevalent disease, there were 262,704 healthy participants that entered the initial state of the Model, based on the distribution observed in GPRD, including 49 % men. There were 37 % aged <45 years and 42 % were aged 45–64 years. All simulations were stratified by single year of age with the initial population aging by 1 year per cycle. Participants exited the model when they died or reached 100 years of age. The model was run for each sex separately. Outcomes and costs were compared for Intervention and Standard Care over 70 annual cycles, this allowed the entire cohort to progress either to death or to reach age 100 and exit the model.

Annual transition probabilities for the model were obtained by sampling from the beta-binomial distribution, using GPRD data as inputs (Table 1). Utilities for each state were obtained from data published in a compendium of values [19] (Table 1). Utility values for each state were stratified by single year of age but were the same for men and women. Utility values were sampled from the beta distribution. The costs of each state were sampled from the gamma distribution with the mean value from GPRD, by 10-year age group, sex, condition and depression status, as the empirical input.

Total costs and quality adjusted life years (QALYs) were obtained by summing across the 70 cycles of the model included in each simulation. There were 2,000 simulations run for each of intervention or standard care scenarios. Results are expressed as rates per 1,000 healthy participants entering the model. Mean costs, and the 95 % range, were obtained from the data for 2,000 simulations. Incremental costs and QALYs were obtained as the difference between intervention and standard care scenarios. Costs and QALYs were discounted using a rate of 3.5 %, but QALYs were also discounted at a rate of 1.5 % as a sensitivity analysis. Incremental costs were plotted against incremental QALYs to present a cost-effectiveness ellipse. Net health benefits (NHB), at a threshold value of £30,000 per QALY, were calculated as the difference between the increment in QALYs and the increment in costs divided by the threshold value of cost per QALY. A cost-effectiveness acceptability curve was plotted using a range of threshold values. The model was implemented with a half cycle correction for the estimation of QALYs and costs.

### Intervention effects

The intervention was assumed to modify only the incidence of disease in healthy participants At Risk. The effect of intervention was estimated as a potential impact fraction (PIF), following Cobiac et al. [20]. The PIF provides a means of estimating the extent to which a change in risk factor exposure is associated with a proportionate decline in the likelihood of an individual developing a disease outcome of interest. The PIF was estimated from three sources of data: (1) the effect of brief interventions in primary care on physical activity levels. Orrow et al. [6] estimated that the number needed to treat for an additional sedentary subject to become active was approximately 12 with an odds ratio of 1.42 (95 % confidence interval 1.17–1.73) and an event rate in control participants of 26 % (507/1924); (2) Data for the distribution of physical activity in the general population, by 10-year age group and sex, were obtained from the Health Survey for England 2008 [21]; (3) Relative risks associating inactivity, or insufficient activity, with the four study disease outcomes (diabetes, coronary heart disease, stroke and colorectal cancer) were obtained from the WHO study ‘Comparative Quantification of Health Risks’ [22]. Consistent with the WHO study we did not include a possible effect of intervention on depression prevalence because the evidence is inconsistent and disputed. The PIF was estimated as outlined by Cobiac et al. [20], with estimates being derived, in each cycle, for single years of age and sex based on the empirical estimates from the three data sources.

The intervention was modelled as being maintained for either 5 or 10 years. In the absence of evidence for the time course of intervention effects, the same estimates were used to model the intervention effect in each of the first five or ten cycles of the model as appropriate.

The cost of the intervention was modelled as a fixed cost per person year depending on their physical activity level. The population At Risk was divided into those that were physically active and those that were physically inactive or who took insufficient physical activity, based on the distribution observed in the Health Survey for England 2008. In the population that was not sufficiently physically active the intervention cost, in the base case, was modelled as being equivalent to the cost of one family practice consultation per person year (£35) [16]. In the population that was physically active, the cost of screening questions to evaluate physical activity levels was 20 % of one family practice consultation per year. Orrow et al. reported that ‘Most [brief physical activity interventions in primary care] included written materials and two or more sessions of advice or counselling on physical activity, delivered face to face’ [6]. As sensitivity analyses, therefore, we also considered costs of intervention in inactive individuals equivalent to two family practice consultations per year (£70) and 20 % of a family practice consultation per year (£7).

## Results

The estimated values for intervention effects, derived from the estimated PIFs, are shown in Table 2. The figures are the mean, standard deviation and range of values for 2,000 simulations, with each value representing the mean across all ages in the first cycle of each simulation. An intervention effect of 0.95 indicates that the incidence of the condition of interest will, on average, be 5 % lower with intervention.

**Table 2:** Estimated values for intervention effects derived from potential impact fractions (PIFs)

	Male	Female
Diabetes mellitus		
Mean (SD)	0.966 (0.010)	0.967 (0.010)
Range	0.919–0.997	0.934–0.998
Coronary heart disease		
Mean (SD)	0.949 (0.015)	0.951 (0.015)
Range	0.892–1.003	0.905–1.001
Stroke		
Mean (SD)	0.968 (0.010)	0.969 (0.010)
Range	0.932–1.001	0.930–1.001
Colorectal cancer		
Mean (SD)	0.959 (0.012)	0.961 (0.012)
Range	0.913–1.002	0.915–1.004

Figures are the mean (SD) and range for 2,000 simulations for values in the first cycle of the model. Values may be interpreted as relative risks

**Fig. 2:**
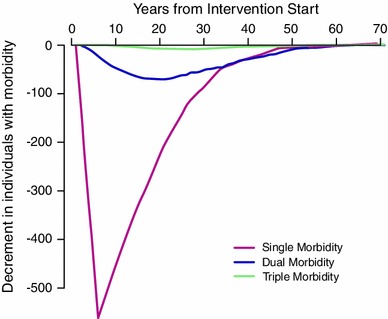
Changes over time in levels of single and multiple morbidity following an intervention of 5 years duration. Data represent the mean difference between intervention and standard care by year (color figure online)

There were 262,704 healthy participants, with the same age and gender distribution as in GPRD, who entered the model in each simulation (Table 3, Fig. 1). For an intervention lasting 5 years, there was an increase in life years lived without physical disease of 52.1 (−10.9 to 115.3) per 1,000 participants entering the model. The probability that life years free from disease was increased was 91.9 %. Figure 2 shows the time-course of changes in single- and multiple-morbidity following the start of intervention. As expected, single morbidities were reduced during the intervention period, but the reduction in single-morbidities persisted, while diminishing, following the end of intervention. There was greater than 95 % probability that single morbidities were reduced from the second to the 11th year following the start of intervention. A reduction in dual morbidities reached its maximum, approximately 20 years following the start of intervention, while a reduction in triple morbidity reached a maximum approximately 30 years following the start of intervention. There was an 87.6 % probability that life years lived with single morbidities was reduced overall (Table 3). The equivalent figure for dual morbidities was 73.3 %; triple morbidities, 58.7 %; and quadruple morbidities, 49.6 %. Although the intervention was modelled to have no direct effect on depression prevalence, overall life years with depression tended to be reduced because of the empirical observation that depression prevalence was higher in individuals with morbidity.

**Table 3:** Health outcomes and cost-effectiveness of a physical activity intervention in a population of 262,704 healthy participants

	Intervention duration			
	5 Years		10 Years	
	Difference (intervention-standard care)	Probability (%)	Difference (intervention-standard care)	Probability (%)
Number entering intervention	262,704		262,704	
Life years lived without disease (per 1,000)^a^	52.1 (−10.9 to 115.3)	91.9^b^	102.3 (42.3 to 163.7)	99.7^b^
Life years lived with physical morbidity (per 1,000)^a^				
Single condition	−34.1 (−82.3 to 13.7)	87.6^c^	−69.6 (−119.3 to −21.6)	98.7^c^
Dual conditions	−8.3 (−29.1 to 12.6)	73.3^c^	−16.1 (−38.8 to 5.7)	88.9^c^
Triple conditions	−0.96 (−8.0 to 5.7)	58.7^c^	−2.0 (−9.0 to 5.0)	69.8^c^
Quadruple conditions	−0.01 (−1.4 to 1.4)	49.6^c^	−0.1 (−1.5 to 1.3)	53.6^c^
Life years lived with depression (per 1,000)^a^	−2.8 (−17.9 to 11.8)	61.9^c^	−6.4 (−20.4 to 7.9)	76.6^c^
Total life years (per 1,000)^a^	8.9 (−35.6 to 52.3)	62.7^b^	14.6 (−29.2 to 59.3)	71.1^b^

Figures represent mean and 95 % range of 2,000 simulations

^a^Per 1,000 healthy participants entering model

^b^Probability measure is higher with intervention

^c^Probability measure is lower with intervention

When the intervention was maintained for 10 years, life years lived free from disease were increased by 102.3 per 1,000 participants entering the intervention (probability increased 99.7 %). Life years lived with single morbidities were reduced by 69.6 per 1,000, probability 98.7 %; dual morbidities were reduced by 16.1 per 1,000, probability, 88.9 %. There was a 69.8 % probability that life years with triple morbidity were reduced and 76.6 % probability that life years with depression were reduced. There was only weak evidence that total life years were increased after either 5 or 10 years intervention (Table 3).

Table 4 shows the changes in costs and QALYs associated with the intervention. With an intervention of 5 years duration, the discounted incremental costs of intervention were £97,572 per 1,000 participants entering intervention, with 86 % of the cost attributable to intervention in participants who were inactive or insufficiently active. Approximately 14 % of the cost of intervention was attributable to confirming the physical activity status of participants that were already active. The costs of non-intervention health care utilisation tended to be reduced through intervention by −£16,818 (probability reduced 63.7 %). Thus the overall total incremental costs under intervention were £80,744 (probability increased 95.5 %).

**Table 4:** Health outcomes and cost-utility of a physical activity intervention in a population of 262,704 healthy participants

	Intervention duration			
	5 years		10 years	
	Difference (intervention-standard care)	Probability (%)	Difference (intervention-standard care)	Probability (%)
Number entering intervention	262,704		262,704	
Intervention costs in physically active (£ per 1,000)	13,995 (13,989 to 14,001)	100.0	24,018 (24,003 to 24,033)	100.0
Intervention costs in physically inactive (£ per 1,000)	83,567 (83,531 to 83,601)	100.0	152,210 (152,110 to 152,306)	100.0
Total intervention costs (£ per 1,000)	97,572 (97,521 to 97,602)	100.0	176,228 (176,113 to 176,340)	100.0
Incremental costs of non-intervention health care utilisation (£ per 1,000)	−16,818 (−94,269 to 60,747)	63.7^b^	−31,760 (−109,077 to 47,599)	74.3^b^
Incremental total costs (£ per 1,000)^a^	80,744 (3,326 to 158,251)	95.5	144,469 (67,103 to 223,843)	99.9
Incremental QALYs (discounted 3.5 %) (per 1,000)	5.9 (−8.2 to 19.7)	75.7	9.8 (−4.6 to 23.6)	87.3
Incremental QALYs (discounted 1.5 %) (per 1,000)	8.6 (−14.7 to 32.4)	72.7	14.9 (−8.7 to 38.5)	85.1
Net health benefits (QALYs per 1,000)	3.2 (−11.1 to 16.9)	64.7	5.0 (−9.5 to 19.3)	72.4
Probability cost effective at £30,000 per QALY (%)	64.7		72.4	

Figures represent mean and 95 % range of 2,000 simulations. *QALY* Quality-adjusted life year, *CHD* coronary heart disease

^a^Per 1,000 healthy participants entering model

^b^Probability reduced

The discounted incremental QALYs associated with intervention were 5.9 per 1,000 participants entering intervention (Table 4). The probability that QALYs were increased among the population was 75.7 %. Net Health Benefits associated with intervention were 3.2 QALYs per 1,000 participants entering intervention. The probability that the intervention would be cost-effective at a threshold of £30,000 per QALY was 64.7 %.

For an intervention maintained for 10 years the overall incremental total costs were £144,469 per 1,000. The incremental QALYs associated with intervention were 14.9 per 1,000 (probability increased 85.1 %). The Net Health Benefits at a threshold of £30,000 per QALY were 5.0 per 1,000. The probability of the intervention being cost-effective at the same threshold was 72.4 %.

**Fig. 3:**
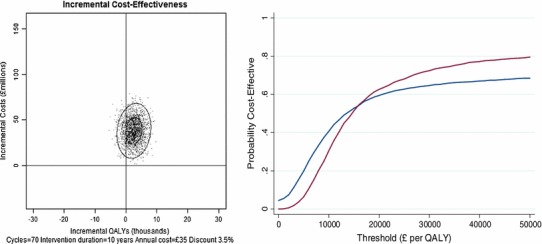
*Left panel* Cost-effectiveness plane showing results of 2,000 simulations with 10 years’ intervention. Outer ellipse encompasses 95 % of simulations. *Right panel* Cost-effectiveness acceptability curves for 5 (*blue*) and 10 (*red*) years intervention (color figure online)

Figure 3 presents a cost-effectiveness plane, in which incremental costs are plotted against incremental QALYs for each of the 2,000 simulations. Figure 3 also presents a cost-effectiveness acceptability curve, in which the probability of the intervention being cost-effective was estimated at different thresholds values of cost per QALY. An intervention continuing for either 5 or 10 years did not achieve more than an 80 % probability of being cost-effective, except at longer intervention duration and high threshold values of cost per QALY, because there were appreciable numbers of simulations in which intervention was associated with no increase in QALYs.

### Sensitivity analyses

Sensitivity analyses were implemented to explore the effects of varying the unit costs of intervention and varying the discount rate. As expected, increasing the unit costs of intervention necessarily made the intervention less cost-effective. If the cost of a 5-year intervention was equivalent to two family practice consultations per year, then net health benefits were approximately zero (−0.06, −14.3 to 13.6 QALYs per 1,000). However, reducing the costs of intervention had only a modest effect because the proportion of simulations in which incremental QALYs were zero or lower set a limit to the potential increase in cost-effectiveness. If the cost of a 5-year intervention was equivalent to 20 % of one family practice consultation per year, then net health benefits were 5.8 (−8.5 to 19.5 QALYs per 1,000, probability cost effective 75.1 %). When QALYs were discounted at 1.5 % rather than 3.5 %, there was only a small difference in the probability of the intervention proving cost effective at 5 years (probability 66.0 %) or 10 years (76.8 %), although estimated mean net health benefits were greater, being 6.0 QALYs per 1,000 for an intervention lasting 5 years and 10.1 QALYs per 1,000 for an intervention lasting 10 years.

## Discussion

### What this study shows

This study modelled the health outcomes of a universal intervention, aimed at all healthy adults, to promote physical activity in primary care. The study employed an empirical population of adults registered with UK family practices; the size of the population at risk was equivalent in size to that of a small town or a primary care organisation. The intervention effect was derived from a meta-analysis of randomised controlled trials, based on the distribution of physical activity observed in a representative population sample in England. The results show that an intervention with only a small effect on the risk of disease may yield appreciable health benefits including an increase in life years lived free from physical disease and a reduction in life years lived with either single or multiple comorbidity. An important finding from this study is that interventions, or intervention effects in terms of behavioural changes resulting from intervention, must be maintained over prolonged periods of time in order for substantial health benefits to accumulate. This long time scale for the emergence of health benefits presents a challenge in allocating primary care resources to delayed rather than immediate benefits. The present results suggest that a brief intervention in primary care represents a costly way of achieving population-wide outcomes. Even when the intervention is delivered at very low unit cost, there is only weak evidence that the intervention could have acceptable cost-effectiveness. These results therefore offer only limited support to continued investigation of a universal intervention as part of a standard family practice visit. Future research should evaluate whether interventions targeted at high-risk individuals may be more suitable for utilisation in primary care, with population strategies being delivered through multi-sectoral interventions.

### What other studies show

Hillsdon’s [23] review of interventions to promote physical activity included studies published up to 2004. Of the 29 studies, 15 were set in primary care, including a range of intervention and follow-up methods. The overall increase in physical activity through intervention amounted to 0.28 standard deviations (SD) for a physical activity measure, with a 0.52 SD increase in physical fitness in 11 studies. This review provides evidence that interventions in primary care to promote physical activity may be effective, at least in the short term. These conclusions are supported by more recent systematic reviews [6, 24]. However, individual level behavioural interventions to promote physical activity may be more costly that we have estimated for this study. Muller-Reimenschneider found a value of Euro 800 per year [25]. Recent studies suggest that community-wide or environmental interventions aimed at increasing use of leisure facilities [26] or promoting active travel [27] may have more acceptable cost-effectiveness.

### Strengths and limitations of this study

This research was grounded in data from a very large cohort of participants from primary care, which provided strong empirical evidence to construct the model. Nevertheless, some multi-disease states were less frequent, leading to imprecise estimates. There were numerous estimates for incidence, mortality, prevalence of depression, as well as health care utilisation contributing to stochastic error in the Model inputs and consequently to uncertainty in the Model outputs. Utility values were drawn from a secondary source because it was not feasible to obtain primary data for multiple disease states within the context of this study. The QALY estimates in this source rely on a US-based survey. While our study is UK-based, this secondary data source provided consistent estimates covering the number of disease states in this research. Uncertainty in utility estimates was incorporated into the Model through the probabilistic approach. Sensitivity analyses were implemented to study the effect of varying key parameters. We did not set an upper age limit to eligibility for intervention because physical activity is beneficial even in old age [28]. The model included only a limited range of health conditions and it is possible that there are wider health benefits of intervention that were not included. The model assumed that mortality reductions would be achieved only through the conditions of interest and not by reductions in mortality from other causes [29]. The model did not include secular trends in the measures of interest because the direction of future trends is unknown.

We acknowledge that true long-term intervention effects are not known and the model requires an important assumption that short-term effects must be maintained if the intervention is continued. We used results from a meta-analysis of randomised trials to provide an estimate of the intervention effect. However, most of the included studies used self-reported measures of physical activity and a similar effect has not been demonstrated employing objective measures. In a probabilistic framework, we used the standard error to model random error in the point estimate of the intervention effect. The intervention time course is also unknown and we did not model scenarios in which the effect of intervention might outlast the intervention itself. We also did not assume any social multiplier effects in our modelling where the impact of one person taking on more physical exercise might influence others’ around him therefore possibly underestimating intervention effect size. Intervention effects were not allowed to vary in different population groups but we intend to study this further in future studies. We modelled several different estimates for intervention costs. It is clear that an intervention of very low cost has only a limited probability of proving cost-effective, with a more costly intervention yielding little or no net benefit. However, more complex scenarios could be envisaged in which the major costs may be incurred at the start, with lower maintenance costs. The model included only health care costs; conclusions might differ appreciably if other costs and productivity changes were to be included. In particular, the opportunity costs of leisure-time physical activity are important [30]. The data were obtained from primary care records and utilisation of secondary care may also have been underestimated as we could not follow patient use of secondary care resources beyond referral and admission. We used the average of costs over all stages of a disease but this is a simplification because, in reality, costs may be higher at the start of the illness, or at periods closer to death. We caution that most estimates included in the Model derived from the British primary health care system. Costs and outcomes may be different in other countries and settings where resource use, the costs of care, and levels of physical activity and barriers to physical activity may be different.

## Conclusions

The results contribute new information towards understanding the potential for a universal strategy for physical activity promotion in primary care. Firstly, an important increase in time lived free from physical disease, and a reduction in the time lived with single or multiple morbidity, may result from even a modest increase in physical activity levels. Secondly, intervention effects must be maintained over a prolonged period of time in order for substantial health benefits to be realised, though these may continue to accumulate after the end of the intervention. Individuals receiving the intervention must effectively change lifelong behaviours in order to benefit. Thirdly, even when interventions can be delivered over the long-term at low annual cost, such as the cost of an additional family practice consultation each year, there is only weak evidence that the intervention might have acceptable cost-effectiveness when employed in a universal strategy. The present results emphasise that physical activity is a determinant of health important to primary care professionals, but also show that implementation of a universal strategy within primary care faces several challenges. While the results indicate some potential for a universal strategy, an alternative approach, which will be evaluated in future research, is the delivery of a selective or targeted strategy to focus intervention efforts on those at higher risk of disease.
